# Dermatophytes adaptation to the human host exemplified by *Microsporum canis*

**DOI:** 10.1080/21501203.2025.2461720

**Published:** 2025-02-16

**Authors:** Xin Zhou, Ricardo Belmonte, Chao Tang, Vania Aparecida Vicente, Sybren de Hoog, Peiying Feng

**Affiliations:** a Department of Dermatology & Allergy, Third Affiliated Hospital, Sun Yat-Sen University, Guangzhou, China; b Department of Medical Microbiology, Center of Expertise in Mycology of Radboud University Medical Center / Canisius Wilhelmina Hospital, Nijmegen, The Netherlands; c Engineering Bioprocess and Biotechnology Graduate Program, Department of Bioprocess Engineering and Biotechnology, Federal University of Paraná, Curitiba, Brazil; d Foundation Atlas of Clinical Fungi, Hilversum, The Netherlands

**Keywords:** *Microsporum*, dermatophytes, comparative genome, adaptive evolution

## Abstract

Dermatophytes are a taxonomic group of keratinophilic fungi that engender cutaneous infections across human and animal populations. The zoophilic species *Microsporum canis*, which exhibits a widespread distribution, predominantly affects domesticated felines and canines and has recently been associated with an increased risk of human adaptation. This study conducted a comparative genome analysis, validating the adaptive expression of 12 relevant genes through neutrality tests and selection pressure analyses, with a particular focus on the evolutionary mechanisms underlying the transition from zoophilic to anthropophilic *Microsporum*. The results demonstrated a high degree of consistency in the nuclear and mitochondrial genomes among the three *Microsporum* species, while significant differences were observed in protein domains. Notably, the anthropophilic species *M. audouinii* and *M. ferrugineum* exhibited more gene duplication events and expansions in domains such as MFS and Zn2Cys6 transcription factors. Among the 138 identified genes, specific protease subfamilies (e.g. S08A, M77, S53) and CAZy subfamilies (e.g. GH18, AA1, AA3) showed strong ecological correlations with either zoophilic or anthropophilic lifestyles. The key functions of these genes from these subfamilies focus on modulating sporulation, endoproteases, lipolysis, pH regulatory adaptability, chitinase, and conidial pigment biosynthesis. Microenvironmental factors such as pH, lipid concentration, and osmolarity significantly influenced the expression of these key genes. Anthropophilic strains demonstrated higher tolerance to acidic pH and enhanced keratinase activity in lipid-rich environments, with *M. ferrugineum* exhibiting the strongest osmotic tolerance. These findings highlight the inherent evolutionary dynamics and adaptive mechanisms of dermatophytes, providing valuable insights into the pathogenicity of *Microsporum*.

## GRAPHICAL ABSTRACT

**Figure:**
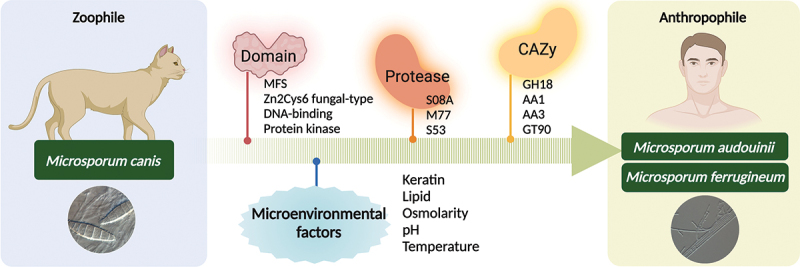

## 1. Introduction

Dermatophytes represent a group of highly infectious fungi that cause superficial cutaneous infections in keratinised tissues in humans and animals, occasionally causing invasive infections. Global prevalence data of dermatophytosis from the World Health Organization indicate a rate of 20%−25% (Havlickova et al. 2008), underscoring the public health problem and substantial economic burden associated with this disorder’s high prevalence, recurrence, and recalcitrance (Kruithoff et al. 2023; Ratemo and Denning 2023). Throughout history, our close association with domesticated animals has played a pivotal role in the epidemiology of dermatophytosis, as evidenced by the fact that more than half of individuals exposed to infected asymptomatic animals may develop symptomatic infections (Moriello et al. 2017; Liu et al. 2024). The wide array of agricultural and companion animal sources of infection has driven repeated evolutionary adaptations of these fungi to the human host. Notably, humans harbour six anthropophilic dermatophytes, a stark contrast to most other mammal species, which typically host only a single or a few species, primarily shared among their phylogenetic relatives (Tang et al. 2023).

Zoophilic *Microsporum canis* is a worldwide diffused dermatophyte that mainly affects pet cats and dogs, and other mammals such as rodents and riding horses, being carried asymptomatically in the fur (Chah et al. 2012). The fungus can be transmitted from animals to human hosts through close contact, leading to clinical symptoms such as tinea capitis and ringworm, sometimes leading to local outbreaks (Ferguson and Fuller 2017). In most cases, human infections by zoophilic dermatophytes tend to be highly inflammatory, while anthropophiles usually cause mild symptoms in the affected host (Wang et al. 2023; Zhou et al. 2023b). However, clinically *M. canis* mainly causes non-inflammatory grey patches suggesting this species is undergoing an ecological transition, which was recently revealed by phylogenetic analyses of host shift of zoophilic entity (*M. canis*) to two sister anthropophiles (*M. audouinii* and *M. ferrugineum*) (Zhou et al. 2023a). Additionally, introduction to new areas and/or adaptation to a new host is probably a unique event in the evolution of different host adaptive dermatophytes, the loss of opposite mating-type partners was an important driver of their evolution, and the successful genotypes that are retained may be significantly dominant in conditions with almost exclusive asexual transmission (Čmoková et al. 2020). Extinction of *MAT1–2* mating-type strains is observed in *M. canis*, but the relatives *M. audouinii* and *M. ferrugineum* were both *MAT1–2*, suggesting adaptation of *MAT1–2* to human hosts (Zhou et al. 2023a). Host shifts have occurred repeatedly during the domestication of agricultural and companion animals and are likely to persist as novel human-animal interactions emerge. Understanding the mechanisms of dermatophyte adaptation is crucial for public health (Fumeaux et al. 2004; Contet-Audonneau and Leyer 2010; Bartosch et al. 2019; Čmoková et al. 2020). The events within the phylogenetically distinct *Microsporum* group serve as an excellent model and can be utilised as a reference for the broader evolution of dermatophytes.

In this study, we conducted comparative genome analysis and transcript-level validation of *Microsporum* in five hosts (horse, rabbit, cat, dog, and humans) and tester strains from various geographic locations, characterising nuclear and mitochondrial genome evolution processes. We compared zoophilic and anthropophilic lineages and performed neutrality tests on 12 potentially relevant orthologous gene sequences. We analysed the adaptive expression of three genes encoding subtilisins in response to microenvironmental changes. Our findings provide important insights into understanding the evolutionary dynamics and the mechanisms of adaptation inherent to *Microsporum*.

## 2. Material and methods

### 2.1. Strains

A total of 13 strains of *Microsporum* species were included in the analysis, with ten strains having undergone genome sequencing and three reference genomes obtained from the NCBI GenBank. The sequenced strains were acquired from the Westerdijk Fungal Biodiversity Institute in Utrecht, Netherlands; the Department of Veterinary Medicine at the University of Bari Aldo Moro in Italy; and the Belgian National Reference Center for Clinical Microbiology at the University Hospital of Liege, Belgium. These strains are maintained at the Center of Expertise in Mycology at RadboudUMC in Nijmegen, Netherlands. Five isolates of *M. canis* were sourced from horses, cats, rabbits, and humans. A pair of sexually reactive tester strains, *M. canis* CBS 496.86 [=VUT 77055 (MT- = *MAT1–1*)] and *M. audouinii* CBS 495.86 [= VUT-77054 (MT+ = *MAT1–2*)] derived from the F1 progeny of two feline isolates (VUT73015 × VUT74001) (Hironaga et al. 1980; Kubo et al. 1987), were included in this study. The remaining three isolates of *M. audouinii* and three isolates of *M. ferrugineum* were sourced from human hosts.

### 2.2. DNA extraction, PCR, and sequencing

DNA was extracted after the strain was incubated in Sabouraud’s Glucose Broth [SGB; 2% (w/v) glucose, 1% (w/v) peptone] at 28 °C, 150 r/min for 3 d. DNA purification was carried out using the QIAquick gel Extraction Kit (Qiagen, Hilden, Germany) according to the manufacturer’s instructions. Genome sequencing was performed using high-quality DNA samples (OD_260/280_ = 1.8–2.0, > 10 µg) on the Illumina Hiseq platforms (Novogene, Milton, England). PCR amplification of proteases and CAZy genes was performed simultaneously on the extracted DNA. Amplicons were verified on 1% agarose.

### 2.3. Genome assembly, annotation, and prediction

The raw reads from the whole genome sequencing (WGS) of ten sequencing samples underwent quality control using FastQC and were subsequently trimmed with Trimmomatic (Bolger et al. 2014). Following the quality control process, the genomes were de novo assembled using SPAdes v3.15.4, as well as gap closing and assembly polishing with GapCloser v1.12 (Luo et al. 2012). The resulting genomes were annotated using the Funannotate v1.5.3 pipeline (https://doi.org/10.5281/zenodo.2604804). Gene prediction was carried out using multiple ab-initio gene predictors, including Augustus v3.5, SNAP v2006-07-28, and Glimme, trained using the fungi orthologous database v10 (Kriventseva et al. 2019) and conserved gene models identified by BUSCO v2 (Simao et al. 2015). Additionally, GeneMark-ES v4.71 was employed for self-training, and Augustus v3.5 was utilised to generate a set of high-quality predictions. The prediction sets were employed for gene structure annotation using EvidenceModeler v1.1.1. The tRNAs were predicted using tRNAscan-SE v2.0.11 (Chan et al. 2021). The gene predictions obtained from EvidenceModeler were annotated using several databases, with hidden Markov model searches PFAM v35.0 and dbCAN v11.0 with hmmersearch v3.2.2 (Mistry et al. 2013), DIAMOND (Buchfink et al. 2021) blastp searches of UniProt DB v2023_02 and MEROPS v12.0, eggNOG-mapper v2.1.11 (Cantalapiedra et al. 2021) searches in diamond mode of the eggNOG 5.0 database (Huerta-Cepas et al. 2019). BUSCO protein searches of the *Onygenales* were done in odb10 with transmembrane and signal peptides being predicted from the proteins using Phobius v1.01 and SignalP v6.0 (Teufel et al. 2022). Antibiotics and secondary metabolite annotation applied antiSMASH v6 (Blin et al. 2021), protein annotation InterProScan5 v5.63-95.0 (Jones et al. 2014), with UniProt/EggNog gene and product names being combined using Gene2Product v1.88. The same gene prediction and annotation procedure was performed for the NCBI reference genomes of *Microsporum* canis (GCF_000151145.1), *M. audouinii* (GCA_022344085.1), and *M. ferrugineum* (GCA_030015455.1).

### 2.4. Mitochondrial genome assembly and annotation

The mitochondrial genomes of the sequenced samples were assembled from the trimmed short reads with GetOrganelle v1.7.7.0 (Jin et al. 2020) using the mitochondrial “fungi_mt 0.0.1” database for read recruitment, with up to 15 recruiting rounds, and multiple kmer values (21, 45, 65, 85, 105) used for the assembly of the recruited reads with SPAdes. The obtained genomes were then annotated using Mfannot (Lang et al. 2023).

### 2.5. Gene orthology and phylogenetic analysis

We utilised OrthoFinder2 (Emms and Kelly 2019) to identify sets of orthologous genes among the analysed strains, employing default settings for DNA sequence analysis. Homology searches were conducted using DIAMOND, gene tree estimation was performed with DendroBLAST (Kelly and Maini 2013), species tree estimation was carried out using STAG (Emms and Kelly 2018), and tree rooting was accomplished using STRIDE. The resulting orthogroups were subsequently employed to investigate differences in specific groups of genes.

### 2.6. Analysis of genetic diversity and selection analysis

Isolation of all orthogroup gene sequences from the proteases and CAZy families differentially enriched in 13 genomes, these orthogroup sequences were aligned using MACSE v2.07 to accommodate frameshifts and stop codons (Ranwez et al. 2011), and then trimmed with TrimAl. Using R v4.3.2, the aligned gene sequences were analysed with the Pegas library v3.1 to determine the average proportion of paired nucleotide differences (π) (excluding insertions/deletions) using the nuc.div function. Nei-Gojobori analyses were performed using the CODEML program of the PAML package to estimate the synonymous and nonsynonymous rates (dS and dN), dN/dS < 1 for purified selection/negative selection, dN/dS = 1 for neutral evolution, and dN/dS > 1 for positive selection. Tajima’s D measures departures from neutral expectations and selection. Tajima’s D neutrality tests using the tajima.test function. For the HKA test, the input information for each locus for the calculation of the HKA test is obtained through SITES, and then the HKA is used to compare the difference between the reference locus and the locus to be detected, and the simulated construction of the distribution gives the statistically significant value of the test (http://genfaculty.rutgers.edu/hey/software). Fst values were estimated using the function “genetic.dist” from the Hierfstat library v0.5–11, with ploidy set to haploid. High Fst values close to 1 indicate strong genetic differentiation between populations, while low Fst values close to 0 indicate homogeneity. Isolation by distance was examined via the Mantel test implemented in Adegenet (Jombart and Ahmed 2011) in R v.3.5.3, the simulated *P* = 0.01. After identification and confirmation of main effect of genomic variants contributing to target traits, DNA markers were developed for molecular assisted selection, within loci linkage-disequilibrium was calculated using the LD function of the genetics package v1.3.8.1.3 (https://CRAN.R-project.org/package=genetics).

### 2.7. Microenvironmental effects

We examined the effects of microenvironmental changes on the transcript levels of *SUBs* in *Microsporum* species. Four factors including lipophilicity, osmolarity, thermotolerance, and pH dependence were assessed. Lipophilicity was evaluated using 0.1%, 0.5%, and 1% Tween-80 in SGB; osmolarity with 3%, 6%, and 9% NaCl in SDB. To assess the growth under acidic and alkaline conditions, SGB was buffered to pH 4.5, 6.5, and 8.5 with sodium citrate or Tris-HCl. *Microsporum canis*, *M. audouinii*, and *M. ferrugineum* were first grown on BLA [SGB; 2% (w/v) glucose, 1% (w/v) peptone] for 10 days, after which conidia/mycelial fragments were eluted from Petri dish with sterilised saline and suspensions at concentrations of (0.5 − 1) × 10^7^ CFU/mL were prepared using the modified EUCAST broken mycelium inoculation method (Risslegger et al. 2015), with four replicates using the above media, three replicates for each strain, and then incubated at 28 °C at 150 r/min for 3 d. Thermotolerance was assessed in SGB at 22 °C, 28 °C, and 37 °C, respectively, shaken at 150 r/min for 3 d.

### 2.8. Real-time quantitative PCR

Real-time quantitative PCR (RT-qPCR) was applied to evaluate the transcript levels of 12 key genes found in this study. Total RNA was extracted using TRIzol (Invitrogen, Waltham, MA, U.S.A.) (Zhou et al. 2024), and first-strand cDNA synthesis was performed using RNA and Transcriptor Universal cDNA Master mix (Roche, Mannheim, Germany). RT-qPCR transcription reactions were performed with the gene primers and LC 480 SYBR Green I Master (Roche). All gene primers used in the manuscript are listed in Table S1.

### 2.9. Statistics and visualization

The data plots generated from the analyses conducted in this study were processed and filtered using the R packages dplyr, ggplot2, GGally, or omicstudio (Lyu et al. 2023), they were refined using Adobe Illustrator v25.3.1. Mitochondrial annotation and comparison were visualised using Genious Prime v2023.2.1. Statistical analysis and plot generation were performed using SPSS v26 and GraphPad Prism v9.0. The two-way ANOVA with Tukey’s multiple comparison test was employed to assess the expression differences.

## 3. Results

### 3.1. *High similarity in nuclear and mitochondrial genomes of* Microsporum *species*

**Figure 1.**
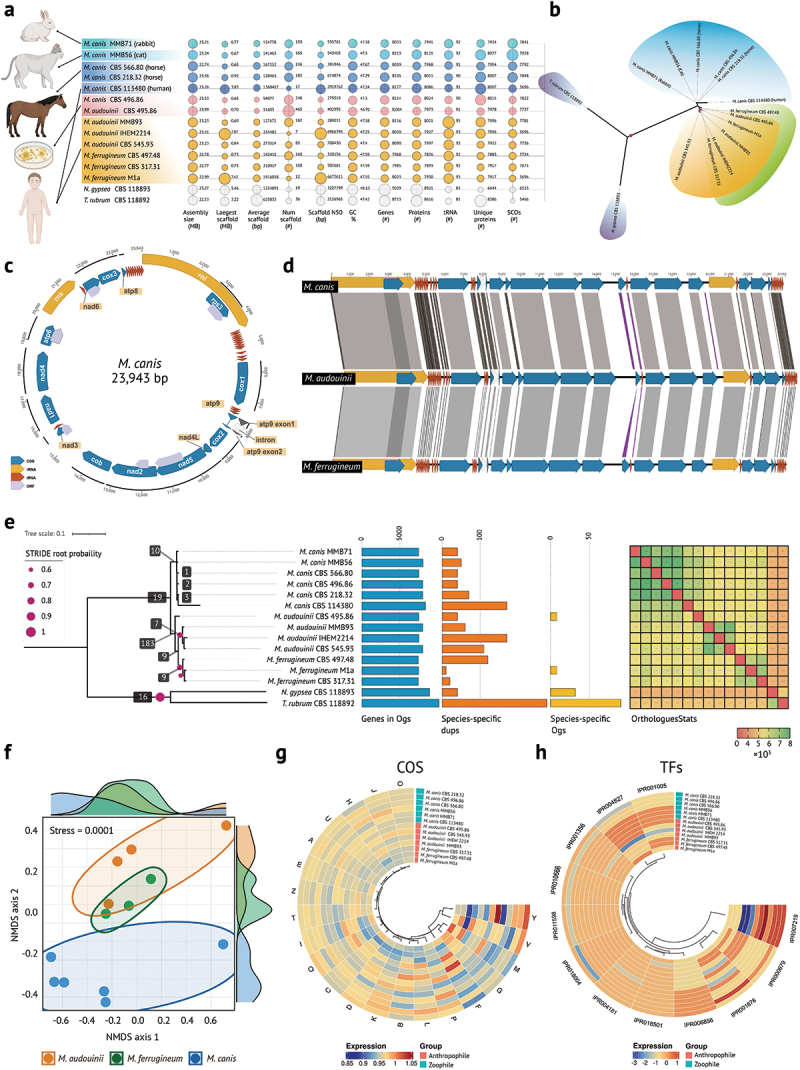
Genome characteristics, phylogenetic orthology inference and homologous protein family analysis. (a) Host origin and genome summary stats for all samples, and bubble plots illustrate the quality parameters of genome sequences. Bubble sizes are not comparable between panels (columns). (b) Unrooted phylogenetic tree inferred by STAG and rooted by STRIDE, the blue, green, and yellow branches represent *Microsporum canis*, *M. audouinii*, and *M. ferrugineum*, respectively. (c) Circular representations of the *M. canis* mitochondrial genomes. (d) Pairwise linear genome comparisons of *M. canis*, *M. audouinii*, and *M. ferrugineum* mtDNAs. Grey parallelograms indicate the locations of homologous genomic regions in adjacent genome pairs. Nine PCGs had length variations among the three *Microsporum* species, with *M. audouinii* having a 12-base extra insertion in the nad6 gene compared to *M. canis* and *M. ferrugineum*, while *M. audouinii* and *M. ferrugineum* had a 135 bp extra insertion in the nad3 gene compared to *M. canis*. (e) Summary of OrthoFinder analysis of *Microsporum* species (including outgroups *N. gypsea* and *T. rubrum*): species tree inferred by STAG and rooted by STRIDE, number of gene duplication events tagged on each node of the species tree; number of genes assigned to orthologous groups for each genome; number of gene duplication events on each terminal branch of the species tree; number of species-specific orthologous groups; heatmap of orthologues per species stats. OG = orthogroup; dups = gene duplication events. (f) NMDS analysis of InterProscan functional predictions for 13 genomes. The vertical coordinate analysis is based on the Bray-Curtis distance matrix. Graph plots and ellipses were generated from the ggplot2 R package implemented using the stat ellipse function. Each dot represents a genome. (g–h) Differences between clusters of orthologous groups (COGs) and fungal transcription factors (TFs) per genome. The plotted values were standardized with normal distribution, distance calculation based on Euclidean, and complete hierarchical clustering. Detailed functions and descriptions of each subcluster are shown in Tables S1–S2.

The reasonable draft genome quality with a total of 13 *Microsporum* strains genomes assembled into fewer than 500 scaffolds (Figure 1a). Genome sizes and gene numbers in animal- and human-derived *M. canis*, as well as in human-derived *M. audouinii*, were comparable and notably greater than those in *M. ferrugineum*. GC percentages were similar in all genomes, ranging from 46.92% to 48.13%. The total numbers of predicted proteins, unique proteins, and tRNAs were similar in three *Microsporum* species. The Benchmarking Universal Single-Copy Orthologs (BUSCO) coverage of all genomes exceeded 98%. Genomic similarity and homozygosity showed a high degree of colinearity, interrupted by a small number of inversions, and a high percentage of identity at the amino acid level (94.11% *M. canis* vs. *M. ferrugineum*; 96.23% *M. audouinii* vs. *M. ferrugineum*; 97.46% *M. canis* vs. *M. audouinii*) (Figure S1). The species tree inferred by STAG showed that the *Microsporum* species was distant from the outgroup (Figure 1b), which was consistent with our previous evolutionary results on conserved sequences, evolutionary change from zoophilic to anthropophilic is observed within *Microsporum*.

The mitochondrial genomes (mtDNAs) of *M. canis* (Figure 1c), *M. audouinii* and *M. ferrugineum* were circular with sequence lengths of 23,943 bp 23,986 bp, and 23,840 bp, respectively, which maintained a high degree of covariance with short intergenic regions and no detectable GC rearrangements (Figure 1d). Similar to other dermatophytes, each *Microsporum* genome encoded 15 core protein-coding genes (rps3, cox1, atp9, cox2, nad4, nad5, nad2, cob, nad3, nad1, nad4, atp6, nad6, cox3, and atp8) and two rRNA genes (rnl and rns). The anthropophilic *M. audouinii* and *M. ferrugineum* encoded 26 tRNAs, encoding an additional trnG (acc) tRNA over *M. canis* (25 tRNAs); these tRNAs were divided into three clusters flanked by rps3-cox1, cox1-atp9, and atp8-rnl. The ribozyme gene (rnpB) was found downstream of the atp6 gene in *M. canis* and *M. audouinii*, which was lost in *M. ferrugineum*.

### 3.2. *Decreasing number of orthologs from animal- to human-derived* Microsporum

The proportion of genes assigned to the orthologous group exceeded 93% for all 13 genomic samples. The ortholog genes were genes derived from species formation events, as opposed to paralogs genes derived from gene duplication events. Based on Heatmap of orthologues statistics for each species pair, the number of orthologs among animal-derived *M. canis* (*n* = 7,759–7,894) was slightly higher than that of human-derived *M. canis* CBS 113480 (*n* = 7,692–7,743), followed by *M. audouinii* (*n* = 7,485–7,710), and *M. ferrugineum* (*n* = 7,439–7,653) (Figure 1e). Among the 13 genomes, only two species-specific orthogroups were found in *M. audouinii* CBS495.86 and *M. ferrugineum* M1a, respectively, and the gene products within these orthologous clusters were all hypothetical proteins. In the species tree inferred by STAG, the number of gene duplication events (support ≥ 50%) on the branch of anthropophilic *M. audouinii* and *M. ferrugineum* (*n* = 183) were significantly higher than that in *M. canis* branch (*n* = 19), suggesting that these strains may have adapted to the host-specific environment through continuous gene duplication (Figure 1e).

### 3.3. Differences in domain function suggest separation of zoophile and anthropophile

We used the SnpEff spacer forest method to compare and annotate genome variants. Two variant virulence factors relating to signal transduction mechanisms and RNA processing were found between human- vs. animal-derived *M. canis*. When comparing human-derived *M. canis* vs. *M. ferrugineum* and *M. audouinii*, the number of potential virulence factors was five and four, respectively. The former functions mainly relate to secondary metabolites, signal transduction, transcription, and intracellular trafficking, while the latter functions relate to cytoskeleton, translation, and cell cycle control (Figure S2, Table S4).

The InterPro, the Gene Ontology (GO), EggNOG, Pfam, and Clusters of Orthologous Groups (COS) database annotations revealed 7,756 (InterPro, 94.85% of gene number), 5,040 (GO, 61.63%), 4976 (EggNOG, 60.85%) and 3,803 (Pfam, 46.51%) function gene predictions, respectively. Non-metric multidimensional scaling (NMDS) ordinations confirmed that zoophilic and anthropophilic *Microsporum* species differed in the distribution characteristics of protein domains (Figure 1f). Zoophile and anthropophile were clearly segregated in composition, with anthropophilic *M. audouinii* and *M. ferrugineum* partially overlapping. There was no segregation between animal- and human-derived *M. canis* strains.

We compared the InterPro category counts of zoophilic vs. anthropophilic *Microsporum* genomes. Of the top 10 differential domains, seven were expanded in anthropophilic *Microsporum* species, with three of them possessing the major facilitator superfamily structural (MFS) (IPR020846, IPR011701, IPR036259), two possessing Zn2Cys6 fungal-type DNA-binding (IPR036864, IPR001138), and two possessing protein kinase (IPR011009, IPR00719). The remaining three were expanded in zoophilic *Microsporum*, carrying P-loop NTPase (IPR027417), WD40-repeat domain (IPR036322), and cytochrome P450 (CPY) (IPR001128) (Figure S3). In comparison of COGs (Figure 1g, Table S1), there were 25 categorised clusters. The major intraspecific differences were centred on (1) carbohydrate transport and metabolism, lipid transport and metabolism, secondary metabolites, and (2) transcription, replication, recombination, and repair.

We further identified transcription factors (TFs), secondary metabolism, secreted proteins, and membrane proteins in *Microsporum* species using the Fungal Transcription Factor Database, antiSMASH 7.0, and Phobius v1.01. The TFs of *Microsporum* species were mainly enriched in the Zn2Cys6 transcription factor (IPR007219), Myb (IPR001005), and basic-leucine zipper (IPR004827), but the differences in the number of TFs among three *Microsporum* species were minimal (Figure 1h, Table S3). There were six secondary metabolite classes involved in the *Microsporum* synthesis: non-ribosomal peptide synthetases (NRPS), T1 polyketide synthases (T1PKS), NRPS-like genes, PKS/NRPS hybrid genes, dimethylallyl transferase, and terpene cyclase. No statistical differences were found in the distributions of secreted protein and membrane proteins in zoophilic and anthrophilic *Microsporum* species.

### 3.4. *Specific proteases and carbohydrate-active enzymes (CAZy) may be involved in* Microsporum *microevolution*

**Figure 2.**
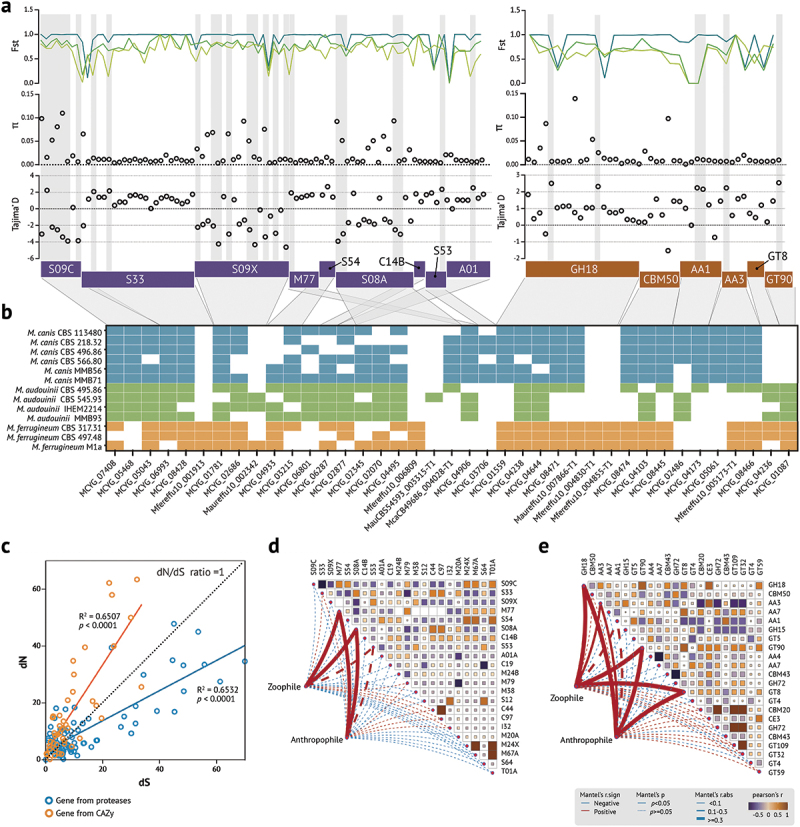
Gene selection for protease and CAZy. (a) A total of 138 genes were isolated from the differential gene families of CAZy and protease and CAZy, showing values of nucleotide diversity, Tajima’s D neutrality test, and Fst statistics for the genetic differentiation between populations in *Microsporum* species. Each black circle represents a gene, Tajima’D and HKA neutrality tests values for genes within the grey rectangle were statistically significant (*p* < 0.05). (b) The Heatmap showing gene loss in samples from different isolation sources. Mfereffu10_001913 (S33, function unknown) and Mfereffu10_006809 (S08A, posttranslational modification) appeared only in *M. ferrugineum*, while MCYG_05468 (S33, function unknown) and MCYG_06801 (M77, coenzyme metabolism) were lost in *M. ferrugineum*. McaCBS49686_004028-T1 (S53, coenzyme metabolism) was present only in animal-derived *M. canis*. Of the CAZy carbohydrates, OG0007899 (AA1, function unknown) and OG0008108 (AA3, secondary metabolites) were only found in *M. canis*, while MCYG_04173 (GT8, carbohydrate metabolism) and MCYG_05061 (GT90, function unknown) are specific to *M. audouinii* and *M. ferrugineum*. (c) Plot of dN/dS ratios for protease and CAZy, each circle represents a gene. (d–e) Correlations between gene families within protease/CAZy and zoophilic or anthropophilic *Microsporum* species were calculated using mantel’s test. Zoophilic for *M. canis*, anthropophilic for *M. audouinii* and *M. ferrugineum*. Mantel’s r statistics is indicated by line width; solid/dashed lines indicate statistical significance, and line colour indicates mantel’s sign.

Based on the above significant intraspecific differences of predicted protein functions in carbohydrate transport and metabolism, we further annotated specific proteases and CAZy among *Microsporum* species using enrichment analysis. Totally, we identified 138 candidate genes which were enriched in 9 protease and 6 CAZy subfamilies (Figure 2a). However, some of the genes were lost in *Microsporum* species of different origins, e.g. in protease, 4 genes were lost in *M. canis*, 5 genes in S08A, S53, and M77 were lost in *M. ferrugineum*; while in CAZy, 10 genes were lost in *M. audouinii* (Figure 2b). We quantified the selection pressures by comparing the dN/dS ratio between zoophilic and anthrophilic *Microsporum* species. In general, most protease subfamily genes belonged to negative selection (dN/dS < 1), while most CAZy positive selection (dN/dS > 1) (Figure 2c). We then computed the genetic differentiation fixation index (Fst), nucleotide diversity (π), Tajima’s D and Hudson-Kreitman-Aguade (HKA) value. A total of 31 selected genes (Tajima’s D value > 2/< −2 and HKA test *p* value < 0.05), including 25 protease and 6 CAZy, were detected. The average Fst between *M. canis* vs. *M. audouinii*, *M. audouinii* vs. *M. ferrugineum*, and *M. canis* vs. *M. ferrugineum* was 0.7361 (range: 0.16667–1), 0.6567 (range: 0.0204–1), 0.9105 (range: 0.11222–1), respectively, which implying the subdivision among three *Microsporum* species (Table S5).

Subsequently, we analysed the above 31 selected genes using the mantel test to evaluate the ecological microevolution between zoophilic and anthrophilic *Microsporum* species (Figure 2d,e). The results showed significant positive correlations were found in three protease subfamilies (M77, S08A, and S53) and five CAZy subfamilies (GH18, AA3, GT90, GT8, and AA1), which contain 12 selected genes (Table 1). In protease subfamilies, the selected genes were *SUB1*, *SUB7*, *PRB1*, *Glip2*, *SedA*, and MCYG_04669, in the CAZy subfamilies, the genes were MCYG_03088, MCYG_06206, *Arb2*, MCYG_08571, MCYG_03226, MCYG_05869.

**Table 1.** Description and annotation of 12 selected genes of proteases and CAZy.

Gene family	Orthogroup	Locus tag		mRNA/product	Annotation	Function in fungi
S08A	OG0003381	MCYG_04495	*SUB1*	Subtilisin-likeprotease 1	Peptidase S8 propeptide/proteinase inhibitor I9	Genes encoding endoproteases of dermatophytes, highly distributed in *Microsporum canis* and *Trichophyton benhamiae*
	OG0004069	MCYG_02345	*SUB7*	Subtilisin-like protease 7	Peptidase S8 propeptide/proteinase inhibitor I9	*Sub7* is the major protease secreted by *Trichophyton* species
	OG0000856	MCYG_02070	*PRB1*	Vacuolar proteinase B	Peptidase S8 propeptide/proteinase inhibitor I9	The *Δprb1* mutants showed reduced aerial hyphae, lower level of sporulation, and a significant reduction in virulence
	OG0006456	MCYG_04669	\	Intracellular serine protease	Peptidase S8, subtilisin-related	Unknown
M77	OG0003906	MCYG_08676	*Glip2*	GDSL esterase/lipase 2	Phosphopantetheine binding ACP domain	Lip2 lipase maintains high activity at low pH in *Yarrowia lipolytica*
S53	OG0007757	MCYG_01559	*sedA*	Tripeptidyl peptidase sedA/sedolisins	Peptidase S53, activation domain	Secreted tripeptidyl peptidase which degrades proteins at acidic pHs and is involved in virulence
GH18	OG0001063	MCYG_03088	\	Chitinas	Chitin-binding, type 1	Unknown
	OG0005920	MCYG_06206	\	Chitinas	LysM domain	Unknown
AA1	OG0003850	MCYG_08539	*Arb2*	Conidial pigment biosynthesis oxidase	Multicopper oxidase-like, N-terminal	Melanin synthesis pathway in *Aspergillus fumigatus* conidia
	OG0003865	MCYG_08571	\	Laccase 1	Multicopper oxidase	Laccase is commonly recognised as the causative agent of opportunistic fungal pathogens
AA3	OG0001123	MCYG_03226	\	Choline dehydrogenase	FAD/NAD(P)-binding domain superfamily	Unknown
GT90	OG0005453	MCYG_05869		DUF821 domain	Glycosyl transferase CAP10 domain	Unknown

### 3.5. *Potential functions and expression of selected genes in* Microsporum

**Figure 3.**
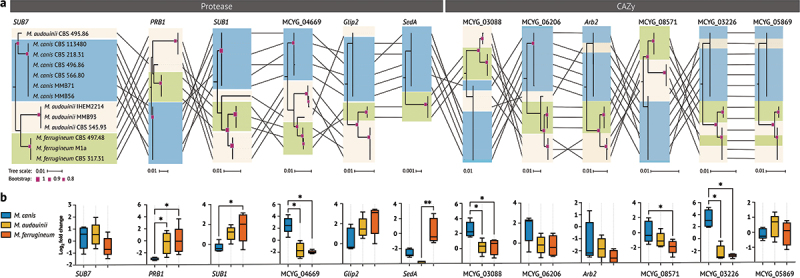
Comparative phylogenetic analysis and key gene expression of protease and CAZy in *Microsporum* species. (a) It shows the tanglegram between the 12 selected genes from protease and CAZy. The black lines connect corresponding parts of the gene tree. Branch lengths are scaled, and pink nodes indicate high-confidence bootstrap values. (b) Boxplots showing the log2 fold changes in the expression of the same genes across the three *Microsporum* species. 5–6 replicates per group and samples from each group are presented using mean ± SEM. Significance was annotated as follows: *p < 0.05; **p < 0.01; ***p < 0.001; ****p < 0.0001.

We performed phylogenetic analyses of the 12 selected genes, and tanglegram plot connected the positional changes of each *Microsporum* species on different gene trees (Figure 3a). The position of the hybrid strain CBS 495.86 in each gene tree was unstable, which might relate to its high rate of nucleotide variation. Proteases genes *SUB1*, *PRB1*, *SUB7*, and MCYG_04669 showed high genetic differentiation for three *Microsporum* species (bootstrap > 90%), while CAZy genes could not distinguish zoophilic *M. canis* from the anthropophilic species. We further analysed the distribution of genetic variants and mutations at SNP sites using the above 12 selected genes. Linkage disequilibrium (LD) analysis revealed highly interlinked blocks for these genes in *Microsporum* species (Figure S4), where the high LD blocks were interrupted by low LD regions, appearing similar to mosaicism in the genome. Notably, a greater number of high LD blocks were detected within genes encoding CAZys, such as MCYG_06206, *Arb2*, and MCYG_08571, and these high local LD could indicate the allele that has recently increased to high frequency under strong selection. We analysed the relationship between SNP sites within each high LD block and phenotype, unique SNPs within Block 1 of *SUB1*, MCYG_03226, and MCYG_04669 were exclusive to *M. ferrugineum*. Similarly, specific SNPs in Block 2 of MCYG_04669, as well as Blocks 1, 2, and 3 of *PRB1*, were solely identified in *M. audouinii*. In contrast, no unique LD blocks were discernible among the different animal-derived *M. canis* strains.

RT-qPCR analysis revealed significant differences in the expression of 12 key genes among the *Microsporum* (Figure 3b). *PRB1* and *SUB1* were significantly upregulated in anthropophilic *M. audouinii* and *M. ferrugineum* (*p* < 0.05), while *SUB7* exhibited no significant expression differences among the three species. *sedA* was significantly upregulated in *M. ferrugineum* (*p* < 0.05), whereas its expression was almost absent in *M. audouinii* and lower in *M. canis*. MCYG_04669 exhibited significantly higher expression in *M. canis* compared to two anthropophilic species (*p* < 0.05). For the CAZy genes, the zoophilic expression levels were higher than those of the anthropophilic species. MCYG_03088, MCYG_08571, and MCYG_03226 were significantly downregulated in *M. audouinii* and *M. ferrugineum* compared to *M. canis* (*p* < 0.05). MCYG_06206 and *Arb2* also show the same decreasing trend in the anthropophilic species but without significant statistical differences.

We further analysed the conserved domains of the protein sequences encoded by these genes and analysed their potential role in *Microsporum*. *SUB1*, *SUB7*, *PRB1*, *Glip2*, *SedA*, and MCYG_04669 whose functions are related to keratolysis, sporulation, lipase, and secreted tripeptidyl peptidase (Table 1). We further analysed the conserved domains of the protein sequences encoded by these genes. In *Glip2*, the anthropophilic *M. audouinii* and *M. ferrugine*um each encode an additional pp-binding conserved domain, with *M. ferrugineum* also possessing an extra PRK0483 domain compared to the other two species. Additionally, the *SedA* gene was lost in the *M. audouinii*, while the other protease genes were largely conserved across all species. In the CAZy subfamilies, MCYG_06206 and MCYG_03088 are known to encode chitinase in dermatophytes. MCYG_06206 consists of two LysM binding, one chitin-binding 1 and one chitin binding domain of the GH18 chitinase family in tandem. MCYG_03088 consists of one LysM binding and one GH18 chitin binding domain. The LysM binding was lost in MCYG_03088 in the rabbit-derived strain *M. canis* MMB71. The *Arb2* and MCYG_08571 genes encode conidial pigment biosynthesis oxidase, with *Arb2* also influencing filamentous growth and spore formation in yeast cells. Laccase 1, encoded by MCYG_08571 gene, is involved in fungal pigmentation and acts as a virulence factor.

### 3.6. Microenvironmental effects on SUB1–SUB3 transcript levels

Subs were core endoproteases for keratin colonisation and infection by dermatophytes. Studies on the pathogenicity of *Microsporum* had proposed that *SUB1–SUB3* were associated with adhesion, keratolysis, and infection of different host hair/keratin proteins. Our previous study of the keratolytic capacity of dermatophytes on 42 species of mammal fur (including wildlife, domestic animals, and companion animals) and phylogenetic analysis revealed that *SUB1–SUB3* genes provide a reasonably correct reflection of the evolution of the *Microsporum* species (Figure S5).

**Figure 4.**
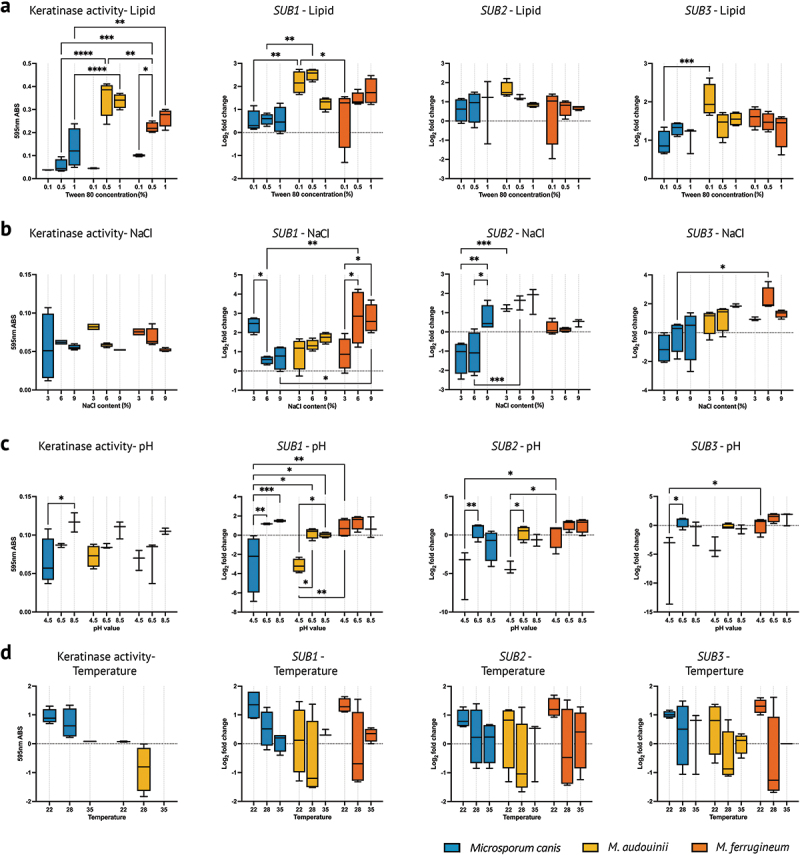
Effects of lipid, osmolality, pH, and temperature on *SUB1–3* transcript levels. Samples from each group are presented using mean ± SEM. The value of *p* < 0.05 was considered statistically significant. Significance was annotated as follows: *p < 0.05; **p < 0.01; ***p < 0.001; ****p < 0.0001.

Corresponding to the ecological microevolution, we further evaluated the microenvironmental adaptation of *Microsporum* species using different concentrations of lipophily, osmolarity, pH, and temperature tolerance factors. As lipid concentrations increased from 0.1%, 0.5% to 1%, the keratinase activity of the three *Microsporum* species gradually increased, most notably in *M. audouinii*, followed by *M. ferrugineum* (*p* < 0.05). At 0.1% and 0.5% lipid concentrations, the expression level of *SUB1* and *SUB3* transcripts of *M. audouinii* were higher than in the other two species (Figure 4a).

We perceived that *M. ferrugineum* strains growing faster than *M. canis* and *M. audouinii* at a high NaCl concentrations of 9%, which suggests that *M. ferrugineum* is more osmolarity tolerant. No significant difference was observed within *Microsporum* species in the effect of changing osmolarity on keratinase activity, but the *SUB1–SUB3* transcript levels of *M. audouinii* and *M. ferrugineum* were higher than those of *M. canis* at 6% and 9% NaCl (*p* < 0.05) (Figure 4b).

In *M. canis*, the expression levels of genes *SUB1–SUB3* were found to be the lowest at pH 4.5, these levels exhibited a gradual increase for *SUB1* across a pH range of 4.5 to 8.5. In contrast, the expression levels of *SUB2* and *SUB3* reached their peak at a pH of 6.5 (*p* < 0.05) (Figure 4c). In anthropophilic *M. audouinii* and *M. ferrugineum*, an increased tolerance to acidic environments was observed, particularly in *M. ferrugineum*, which displayed a marked increase in the expression levels of *SUB1–SUB3* genes at pH 4.5 when compared to *M. canis* (*p* < 0.05).

Alterations in temperature exerted minimal impact on the keratinolytic capabilities and the expression levels of the *SUB1–SUB3* genes across all *Microsporum* strains evaluated. Statistical analysis revealed no significant differences in these parameters among the various temperature conditions tested 22 °C, 28 °C, and 35 °C (Figure 4d).

## 4. Discussion

Dermatophytes (family *Arthrodermataceae*) are a group of fungi in the order *Onygenales* which have evolved relatively recently, almost exclusively with mammal hosts (Kandemir et al. 2022). The ecological subdivision in geophilic, zoophilic, and anthropophilic species broadly reflects their course of evolution, although the molecular mechanisms and evolutionary dynamics driving host shift are still not well understood. *Homo sapiens* is phylogenetically the most recent host, in spite of dermatophyte genomes usually showing strong colinearity and low inversion (de Hoog et al. 2021; Kabtani and Ranque 2023). In *Trichophyton* genomes, the closely related siblings *Trichophyton rubrum* (anthropophilic, preferences nail/skin) and *T. violaceum* (anthropophilic, scalp) have high degrees of genome identity (99.83%), while with marked differences in phenotypic and clinical features, which possibly due to the functional differences in specific proteins involved ATP transferase activity, calcium binding and lipid transport (Zhan et al. 2018). Martinez et al. (2012) demonstrated that anthropophilic species (*T. tonsurans* and *T. rubrum*) were enriched in kinases and transcription factors compared to zoophilic species (*T. verrucosum*, *T. equinum*, *M. canis*, and *T. benhamiae*). In *Microsporum* genomes, zoophilic *M. canis* and anthropophilic *M. audouinii* and *M. ferrugineum* exhibit a high percentage of amino acid identity and show minimal variation in both nuclear and mitochondrial genomes. However, through our phylogenetic inference of orthologous groups on pairwise relationships of the three species, NMDS analyses based on protein domain, and population fixation index (fst) results, all suggest that *M. canis*, *M. audouinii*, and *M. ferrugineum* have diverged significantly in terms of protein function, anthropophilic *Microsporum* species display an expansion of major facilitator superfamily (MFS) and kinase-like domains, while protein families affecting ATPase post-translational modification, signalling mechanisms, and energy production and conversion are contracted, presumably linked to the low inflammatory response to human dermatomycosis caused by anthropophilic *Microsporum* species. Notably, an enrichment of CPY in *M. canis* is observed for the first time. In the newly identified dermatophyte species *T. inotineae*, the overexpression of TinCYP51B resulting from additional copies of this gene is responsible for the reduced sensitivity of *T. indotineae* strains to azole compounds (Yamada et al. 2022). It is reported that 25%–40% of *M. canis* infection patients had recurrence and treatment failure. The role of CYP in the azole resistance of *M. canis* requires further verification.

The ability of microorganisms to sense and adapt to changes in the environment is essential to their survival. Dermatophytes are a highly specialised group of keratinophilic and keratinolytic filamentous fungi. Anthropophilic and zoophilic dermatophytes adapt to different mammalian body surface microenvironments such as temperature, pressure, salinity, and pH by developing molecular mechanisms which stabilise their proteins, cell membranes, lipids, and nucleic acids. The secreted proteolytic and lipolytic enzymes enable nutrient acquisition from the host, leading to variation in host specificity, immunogenicity, and virulence. Transcription factors such as *PacC* and *Hfs1*, as well as heat shock proteins, are involved in sensing and adapting to the acidic pH of the skin in the early stages of fungal host interaction (Ferreira-Nozawa et al. 2006; Martinez-Rossi et al. 2016). Dermatophytes adhere to keratinocyte surfaces in a pH-dependent manner, and the acidic pH of the human skin surface is associated with specific fatty acids derived from skin lipids. Keratin is the only source of carbon for dermatophyte infections, and the keratolytic is accompanied by a change in extracellular pH from acidic to alkaline, an environment in which most of the known proteases exhibit optimal activity, ultimately leading to the establishment and maintenance of the infection (Martinez-Rossi et al. 2017). In our study, we found that subtilisins (*SUB1*, *SUB7*, *PRB1*), lipases (*Glip2*), and tripeptidyl peptidase (*SedA*) were associated with host selection preferences in *Microsporum* species, the latter two genes maintained high activity at acidic pH. Likewise, putative GDSL lipase (*Glip*), subtilisins (*SUB3* & *SUB7*), and tripeptidyl peptidase *SedC* were also observed in the secretomes of *T. benhamiae*, *T. rubrum*, and *T. violaceum* after incubation with keratinocytes (Burmester et al. 2011). According to the MEROPS peptidase classification, subtilases were divided into two clans: S08A (subtilisins) and S53 (sedolisins) (Muszewska et al. 2011). *SedA* (from S53 subfamily) was absent in *M. audouinii*. In *Onygenales*, the lack of certain lineages encoding S53 family genes is thought to be more specific, may play an important role in interactions with the environment (Muszewska et al. 2011). Subtillisins are essential proteases in keratin assimilation, and subtilisin-like protease *SUB1*, *SUB3–SUB7* are specific for dermatophytes, although the expression of *SUBs* gene is not uniform for all species (Descamps et al. 2002; Vite-Garin et al. 2024). In this study, different keratinase activity and expression of *SUB1–SUB3* in response to microenvironmental changes were observed, which was comparable with our previous study (Zhou et al. 2024). The presence of lipids in the environment significantly impacted the keratinase activity of anthropophilic species. Human scalp pH (4.5–5.5) is more acidic than skin pH (6.5–8.5) in pet cats and dogs; high *SUB1–SUB3* transcript levels of *M. ferrugineum* at acidic pH suggests adaptation of the anthropophilic to human hosts. *PRB1* is a subtilisin-like protease essential for virulence and phenotypical traits in many fungi. Its elevated expression in *M. ferrugineum* and *M. audouinii* may enhance colonisation and persistence in human hosts. Interestingly, *sedA* (MCYG_01559), a tripeptidyl peptidase from the S53 subfamily that degrades proteins at acidic pH, is nearly absent in *M. audouinii* but significantly upregulated in *M. ferrugineum*, indicating lineage-specific adaptation to acidic skin environments in *M. ferrugineum*.

We observed an expansion of carbohydrate metabolism-related superfamily in the anthropophilic *Microsporum*, from which we isolated four subfamilies associated with the adaptive evolution of CAZy: GH18, AA1, AA3, and GT90, some of which encode unknown functional domains. Within the GH18 family, we identified MCYG_03088 and MCYG_06206, which encode chitin-binding and LysM-binding, respectively. The LysM domain is a highly conserved carbohydrate-binding module. Compared to other non-dermatophytes in *Onygenales*, the LysM protein family of dermatophytes appears to have undergone a gene expansion, ranging from 7 in *E. floccosum*, 12 in *T. rubrum*, and 31 in *M. canis* (Martinez et al. 2012; Persinoti et al. 2018). Studies have shown that the dermatophyte LysM proteins bind to and mask chitin to protect the fungal cell from the host immune system, allowing latent colonisation by the dermatophyte (Kombrink and Thomma 2013; Kar et al. 2019). In the *Onygenales* phylogenetic tree of all proteins containing LysM domains (Martinez et al. 2012), MCYG_03088 and MCYG_06206 of *M. canis* were clustered in two distinct branches, and further studies should be conducted on the expression and determination of their putative roles in the infection process. The conidial pigment biosynthesis oxidase encoded by *Arb2* and laccase 1, encoded by MCYG_08571 from the AA1 family, are structurally homologous, both containing three copper-oxidoreductase domains. They have been shown to be key regulatory genes for conidial pigment biosynthesis in *Aspergillus fumigatus* and *Blastomyces dermatitidis*, which are involved in cell wall formation and help the fungi resist various environmental stressors (Chang et al. 2020). In the human host, *A. fumigatus* conidial pigments modulate the host’s pro-inflammatory cytokine response by physically masking fungal pathogen-associated molecular patterns (PAMPs) from recognition by the immune system (Liu et al. 2021). Our RT-qPCR results showed that MCYG_03088, *Arb2*, MCYG_08571, and MCYG_03226 were significantly downregulated in anthropophilic *Microsporum* compared to the zoophilic *M. cani*s. Further investigation is required to determine whether these genes serve similar functions as above mentioned in *Microsporum* species. Furthermore, *M. ferrugineum* exhibited greater osmotic stress tolerance compared to the other two species and maintained consistent transcript levels of *SUB1* and *SUB3* even at high NaCl concentrations. This suggests that *Arb2* and MCYG_08571 genes may play an important role in exerting the adaptive evolution of anthropophilic species to human hosts.

## 5. Conclusions

This study represents the inaugural comprehensive genomic sequencing analysis of the different host-derived *Microsporum* species, which, as a phylogenetic group, are clearly individualised (de Hoog et al. 2017) and show a host shift from animal to human. Carbohydrate metabolism, signal transduction regulation, and post-translational modifications were implicated in the microevolutionary processes that underpin host specificity. Notably, there is an expansion of the MFS, Zn2Cys6 DNA-binding, and protein kinases within the anthropophilic *Microsporum* species, with less variation among different animal-derived *Microsporum* species. Specific subfamilies of proteases (M77, S08A, and S53) and carbohydrate-active enzymes (GH18, AA3, GT90, GT8, and AA1) display positive correlations with ecological parameters to anthropophilic and zoophilic lifestyles. We have pinpointed 12 genes within these subfamilies that are pivotal for attributes such as phenotype, sporulation, endoproteases, lipases, and those involved in pH-adaptive regulation. The study further reveals the keratinolytic potential and the expression levels of *SUB1–SUB3* genes are modifiable by lipid, pH levels, and osmolarity in microenvironment. In addition, we identified highly interlocked Block regions in coding protease and CAZy genes and analysed the relationship between SNP site mutations and haplotype, which provides a basis for efficient identification of *Microsporum* species and accelerates mining of functional genes.

## Supplementary Material

accept-Supporting Information.docx

## Data Availability

All new genomes published in the study have been deposited in GenBank under the following BioProject accession number PRJNA1102285. All software used were from publicly available sources.

## Abbreviations

MAT: Mating-type
SGB: Sabouraud’s glucose broth
SUBs: Subtilisins
BUSCO: Benchmarking universal single-copy orthologs
STAG: Species tree inference from all genes
GO: The gene ontology
COS: Clusters of orthologous groups
NMDS: Non-metric multidimensional scaling
TFs: Transcription factors
CAZy: Carbohydrate-active enzymes
HKA: Hudson-Kreitman-Aguade
Fst: Fixation index
Hap: Haplotype
LD: Linkage disequilibrium
MFS: Major facilitator superfamily
