# Supplementary material for: Dermatophytes adaptation to the human host exemplified by Microsporum canis
Source: Mycology. 2025 Feb 16;16(3):1357–72. doi: 10.1080/21501203.2025.2461720 (PMC12422044; doi:10.1080/21501203.2025.2461720)
Supplement: accept-Supporting Information.docx [file TMYC_A_2461720_SM3805.docx]

**Supporting Information**

**RESEARCH ARTICLE**

**Dermatophytes adaptation to the human host exemplified by *Microsporum canis***

Xin Zhou^a,b^, Ricardo Belmonte^c^, Tang Chao^b, d^, Vania Aparecida Vicente^c^, Sybren de Hoog^b,d^*, Peiying Feng^a^*

^a^Department of Dermatology & Allergy, Third Affiliated Hospital, Sun Yat-Sen University, Guangzhou, China

^b^RadboudUMC/CWZ Center of Expertise for Mycology, Nijmegen, The Netherlands

^c^Engineering Bioprocess and Biotechnology Graduate Program, Department of Bioprocess Engineering and Biotechnology, Federal University of Paraná, Curitiba, Brazil

^d^Foundation Atlas of Clinical Fungi, Hilversum, The Netherlands

*Corresponding authors:

Sybren de Hoog [sybren.dehoog@radboudumc.nl](mailto:sybren.dehoog@radboudumc.nl)

RadboudUMC/CWZ Center of Expertise for Mycology, Nijmegen, The Netherlands

Peiying Feng [fengpy@mail.sysu.edu.cn](mailto:fengpy@mail.sysu.edu.cn)

Department of Dermatology & Allergy, Third Affiliated Hospital, Sun Yat-Sen University, Guangzhou, China

**
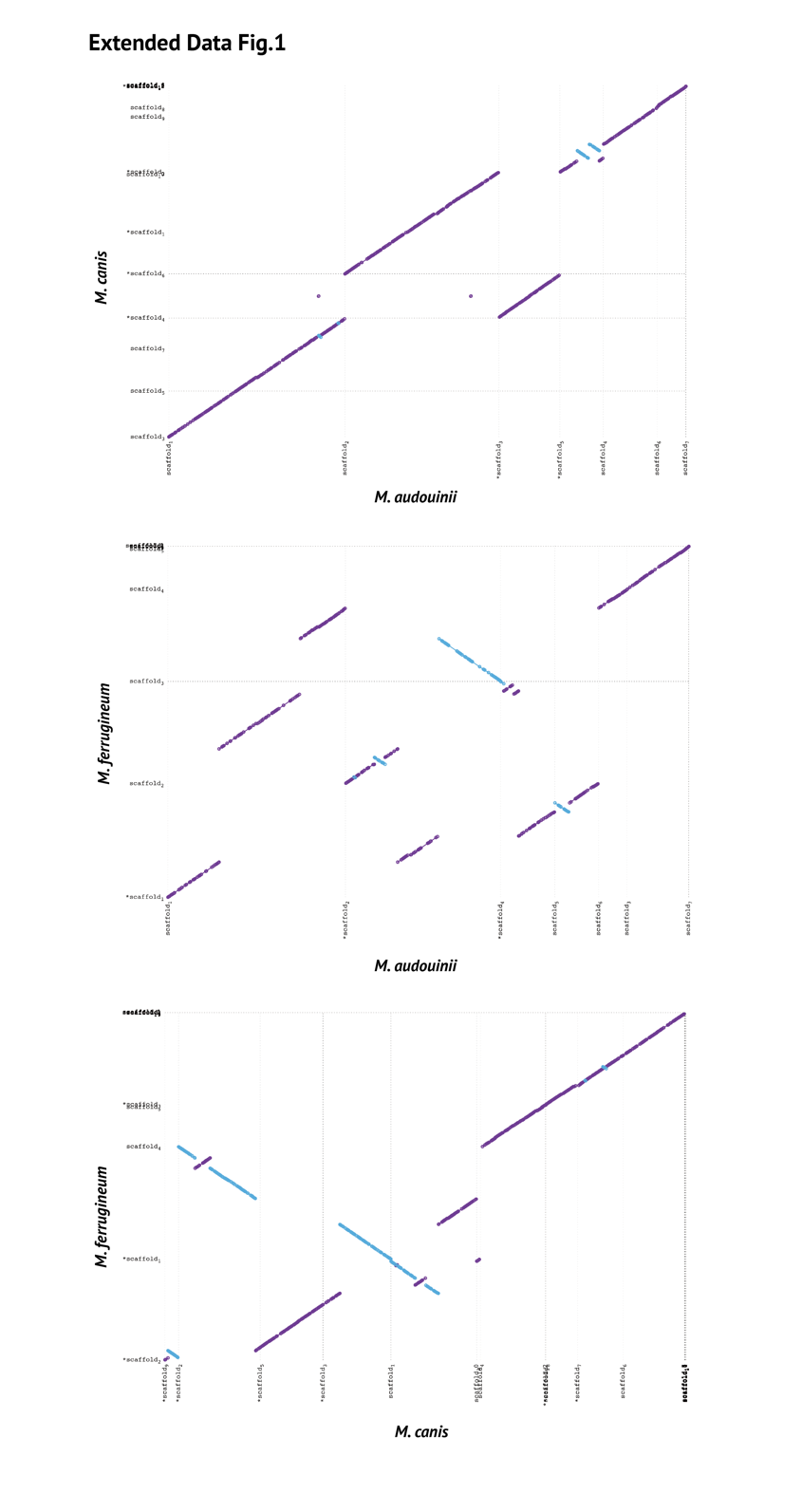
**

**Figure S1.** Alignment of three *Microsporum* species genome using MUMmer4.0 beta.

The dotplot of all the MUMs between two sequences can reveal their macroscopic similarity. Purple lines/dots represent an undisturbed segment of conservation between the two sequences, blue lines/dots represent an inverted segment of conservation. The closer a plot is to a line f(x) = x (or -x), the fewer macroscopic differences exist between the two sequences.





**Figure S2.** Genetic variant annotation and functional effect prediction.

To analyze the variation within species, strain data were mapped to the NCBI reference genomes with BWA using the MEM algorithm (Li 2013). Using GATK v4.4.0.0, these were screened for duplicates with MarkDuplicates default settings, and used for haplotype calling with the HaplotypeCaller, with the following non-default parameters: “-pair-hmm-gap-continuation-penalty 10, -stand-call-conf 30 --sample-ploidy 1”. The resulting vcf files and the reference genomes were used to annotate and predict variant effects using SnpEff v5.1d (Cingolani et al. 2012). The top ten genes in terms of variation frequency were annotated and the genes of the target species and their corresponding functional annotations were compared with the DFVF: Fungal Virulence Factor database Sankey plots of genomic variants were created using SankeyMATIC (<https://sankeymatic.com/>). The width of the grey mobility lines between panels represents differences in the number of variants. Based on the classification and number of variants, the genes with the greatest mutational differences between the three species were compared, and their functions were annotated. Locus tag/name is based on the annotation of the *Microsporum canis* CBS 113480 reference genome.

**
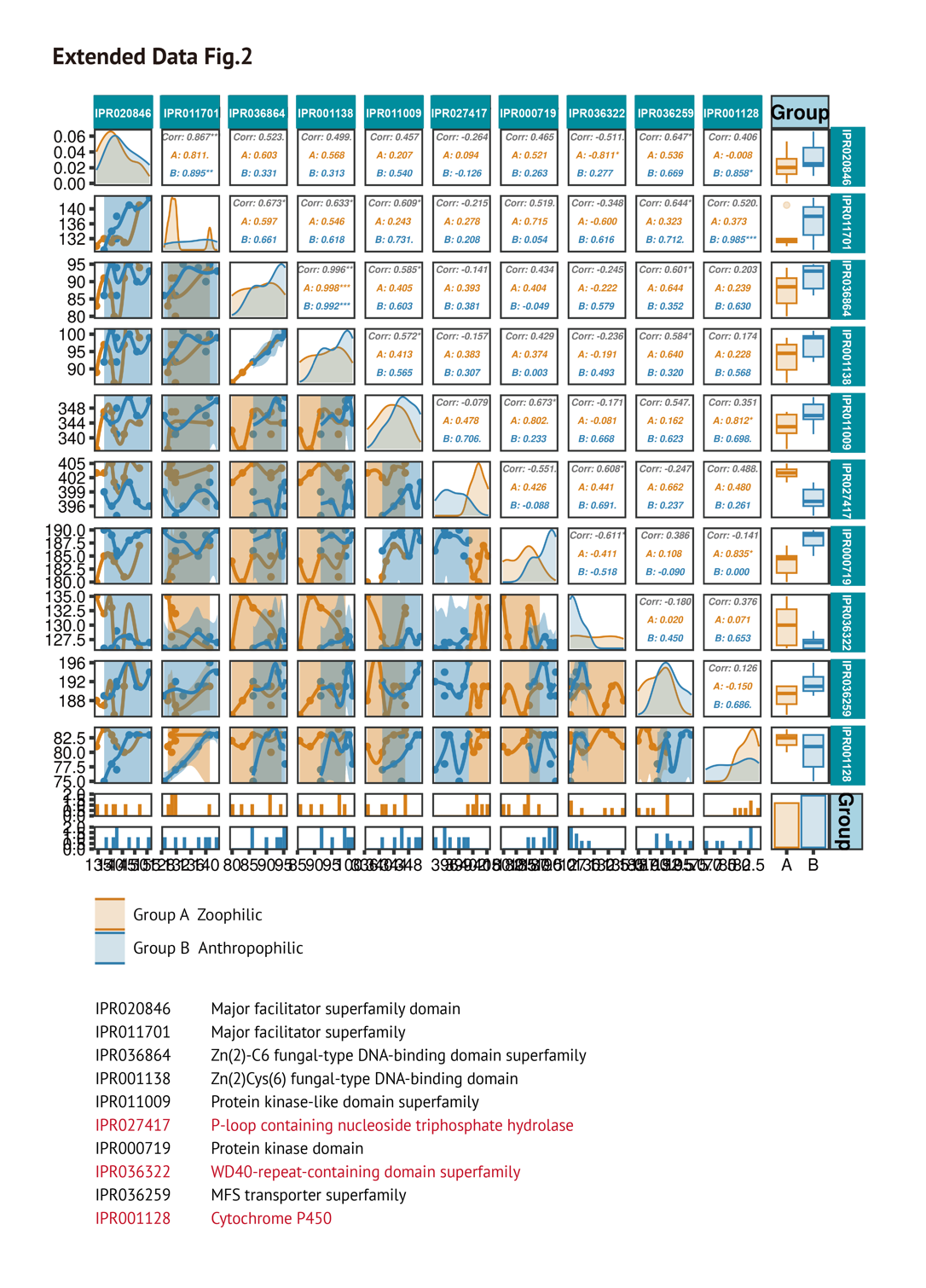
**

**Figure S3.** Data characteristics and correlations matrix between major differential protein domains in anthropophilic and zoophilic *Microsporum* species.

Complex correlation matrix plots are drawn using the R package GGally. Using KendaII for correlation analysis style, the top diagonal plot area shows the correlation coefficient and p value, the * represents the significance level. The diagonal plot area shows the density map corresponding to each domain, and the X-axis position where the peak appears indicates that the data of the domain is mostly concentrated in this range. The lower diagonal plot area shows the scatter plot between two domains, and the curve is smoothed using loess.





**Figure S4.** Linkage disequilibrium analysis.

Figure S4 is a visualization of the LD blocks for each of the variant loci of these genes. The colors represent R^2^ values, with 0 to 1 overriding from blue to orange. Gene models are shown at the top of the figure, with lines representing genomes, gray rectangles representing exons, and different colored circles representing strain variants occurring at the SNP locus. Color-bonded LD blocks are located at the bottom. Strongly linkage Block regions are marked by thick black triangles.


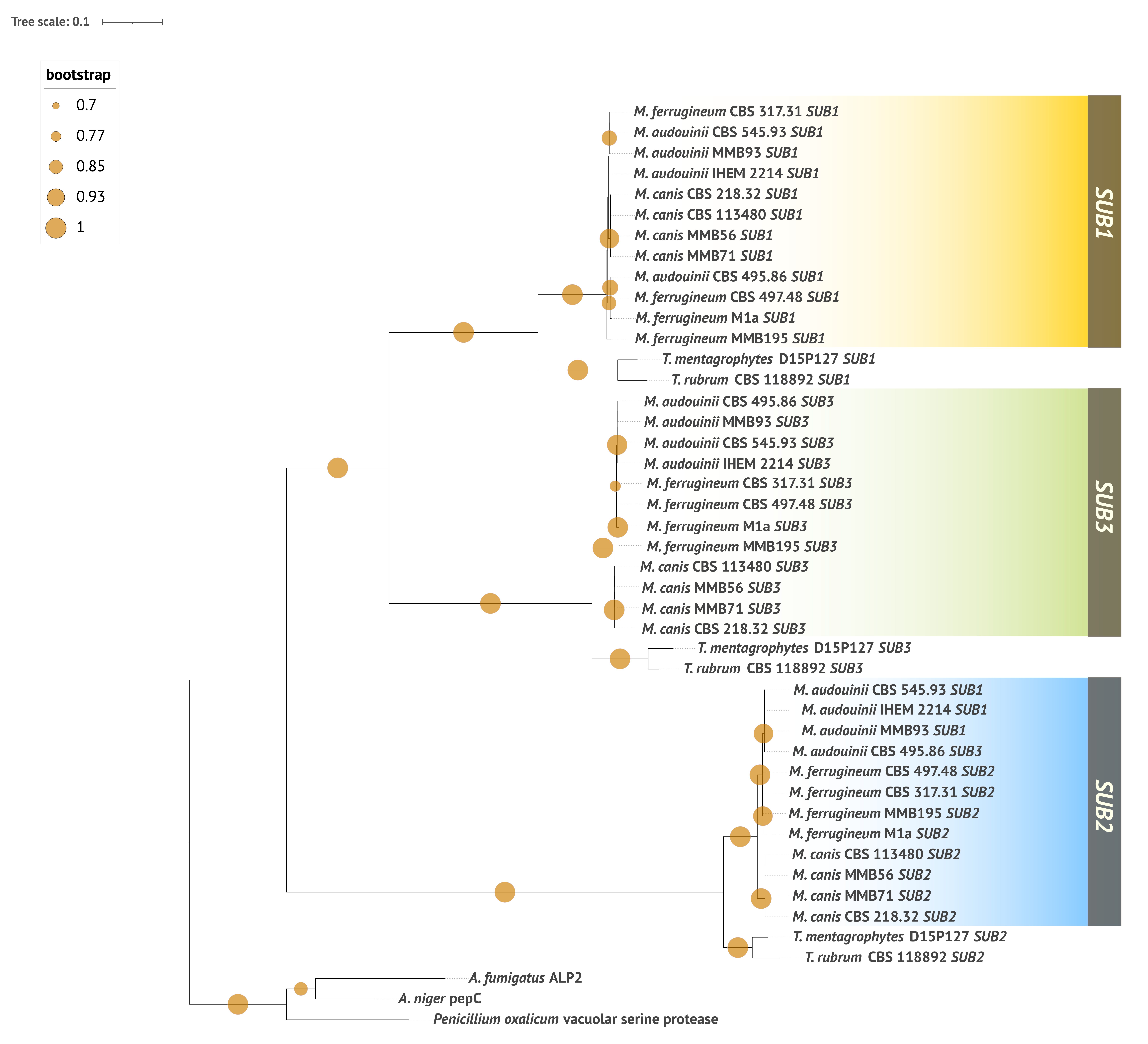


**Figure S5.** Phylogenetic tree constructed from *SUB1-SUB3* genes. *Aspergillus fumigatus, A. niger,* and *Penicillium oxalicum* as outgroups.

**Table S1.** Primers.

| **Locus tag/name** | **Forward Primer（5’-3’）** | **Reverse Primer（5’-3’）** |
| --- | --- | --- |
| *Actin* | CGAGCGTGGCTACAGCTTCT | CTCCTTGATGTCACGGACGAT |
| *SUB1* | TACTCACAACGTTACCCGCC | GTTGGAACCAGGAGCGAAGA |
| *SUB2* | TCCTCACGTTTGTGGAGTCG | ACTTTCCAGTGCGAGCTGTT |
| *SUB3* | CATCAAGCAGATCGCTAATGCC | CACTTCCGTTGTAGAGGAGCTT |
| *SUB7* | CGGTGGTGTCTTCCTAGCTG | GACAGTGCAGATGGATGGCT |
| *PRB1* | GGGTAACCGAAAGGGCTTCA | TGGTCAACAACACGGTCCAA |
| MCYG_04669 | GAATCGGGAGCAACCAGACA | GTTTGCCTTTGACCTCGCTG |
| *Glip2* | CAGCCTGATTGATGGGTGGT | GTGAACTGTGGCGGTAGTGA |
| *sedA* | CCAGCCAACGGTAGTGTTCT | GTTTAGGTTGACGGGGAGGG |
| MCYG_03088 | TATGGCGTTGCCTACCGTTT | GAGTGGCACAGCCATCTTCT |
| MCYG_06206 | GTCTCTGGGTGGGTGTCAAG | CGCCCTTTGGAGGTATTGGT |
| *Arb2* | CGGCGCAATGTACGAAGAAG | AACTCCTGGGGCTGATTTGG |
| MCYG_08571 | CTGGCTCGAAGGACCCTAAC | CAACGGCAATCACCTTGAGC |
| MCYG_03226 | ATATGGTGGGAGGCTGGTCT | CTTCTCTACCATCCACGCCC |
| MCYG_05869 | ACGAAAGCGGAGCAAGAAGA | GTCACGTACTTCCAAGCCCA |

**
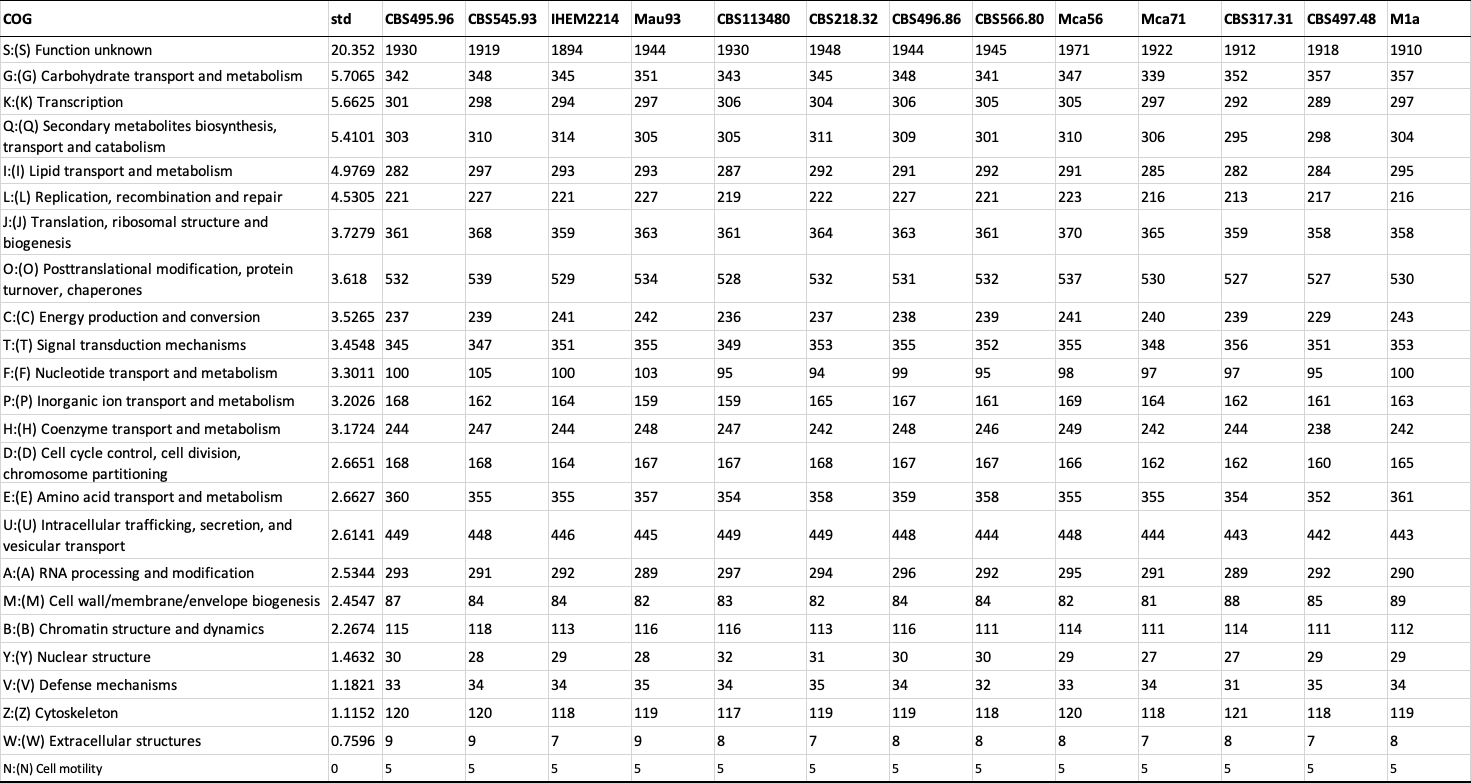
Table S2.** Clusters of orthologous groups (COG) functional category distribution among *Microsporum.*

**
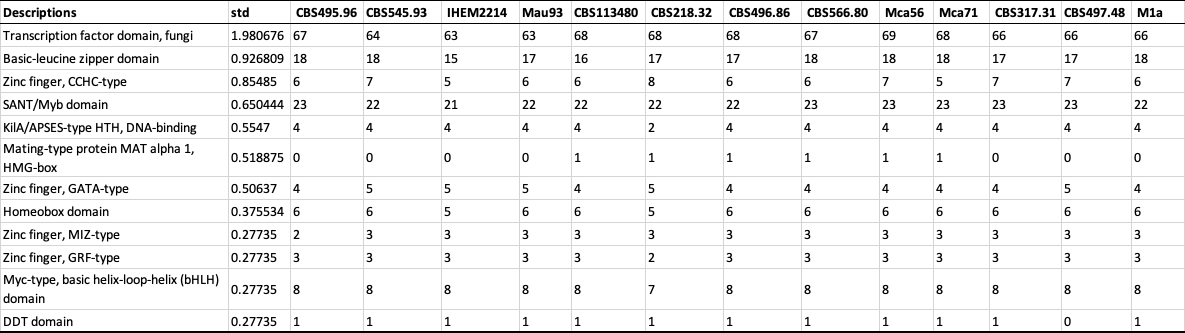
Table S3.** Transcription factors (TFs) functional category distribution among *Microsporum.*

**
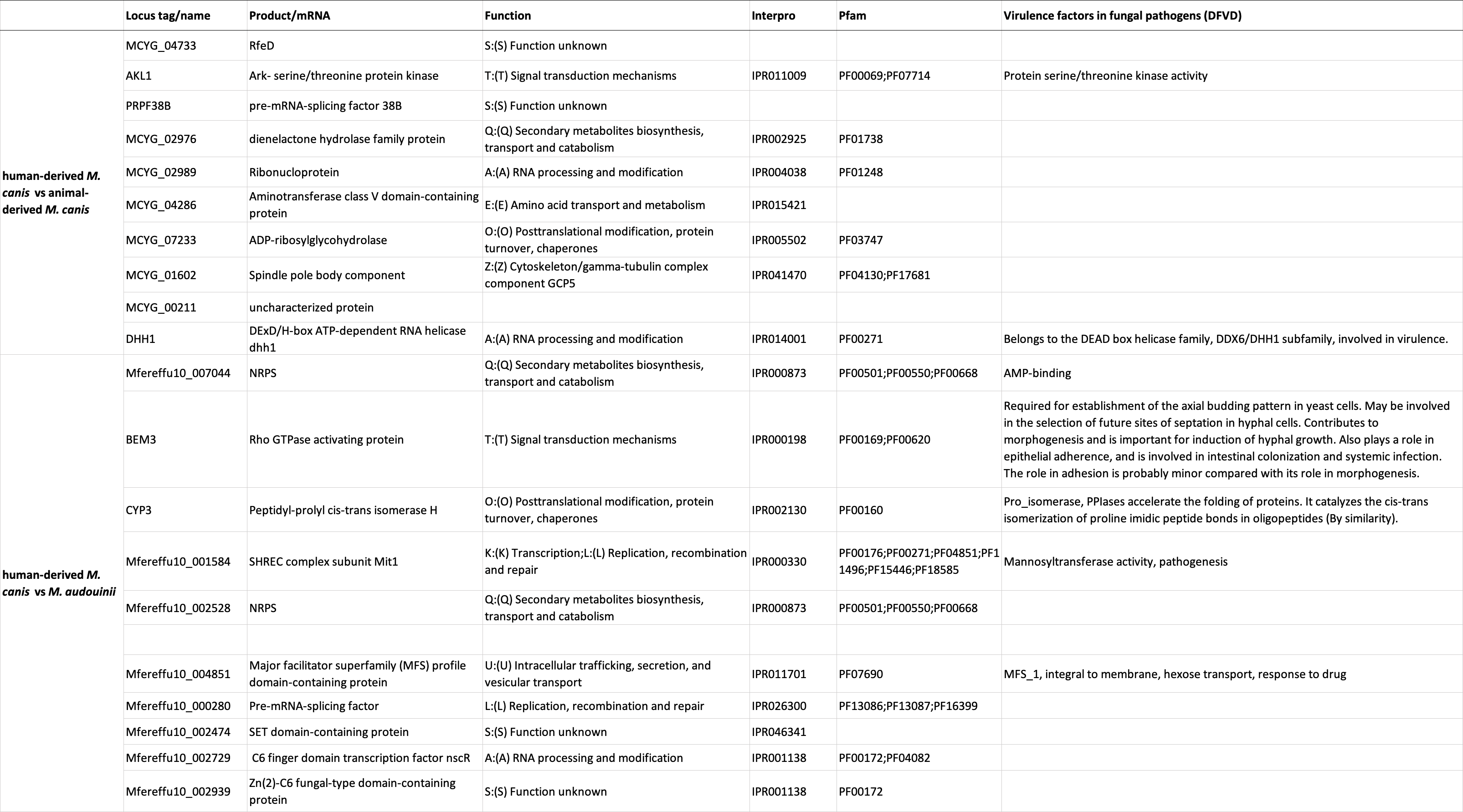
Table S4.** Functional annotation and virulence factors of differential genes in human- and animal-derived *Microsporum* species.


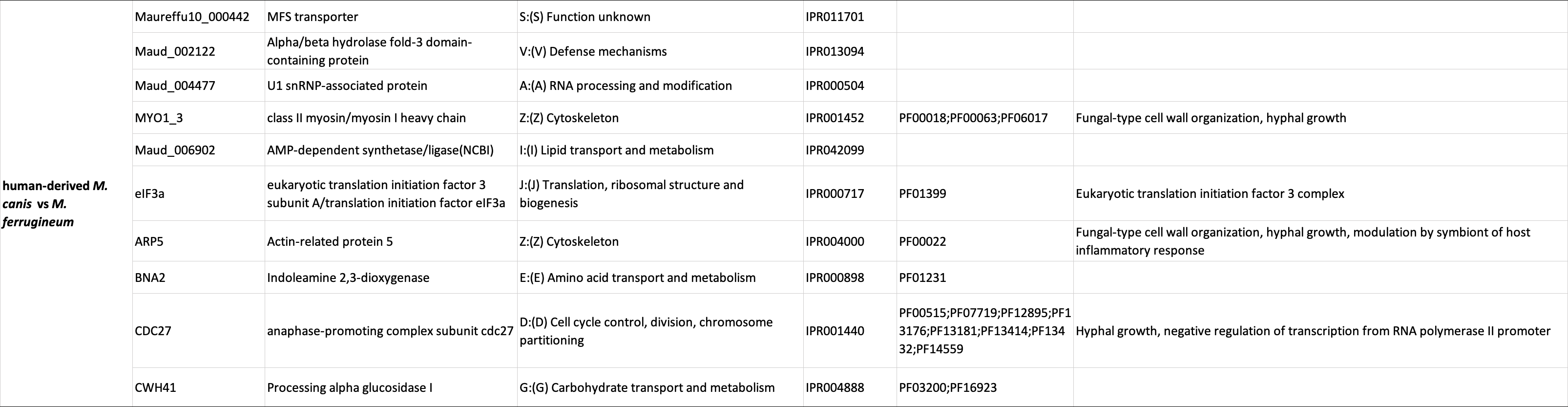
**Table S4.** (Continued).

**
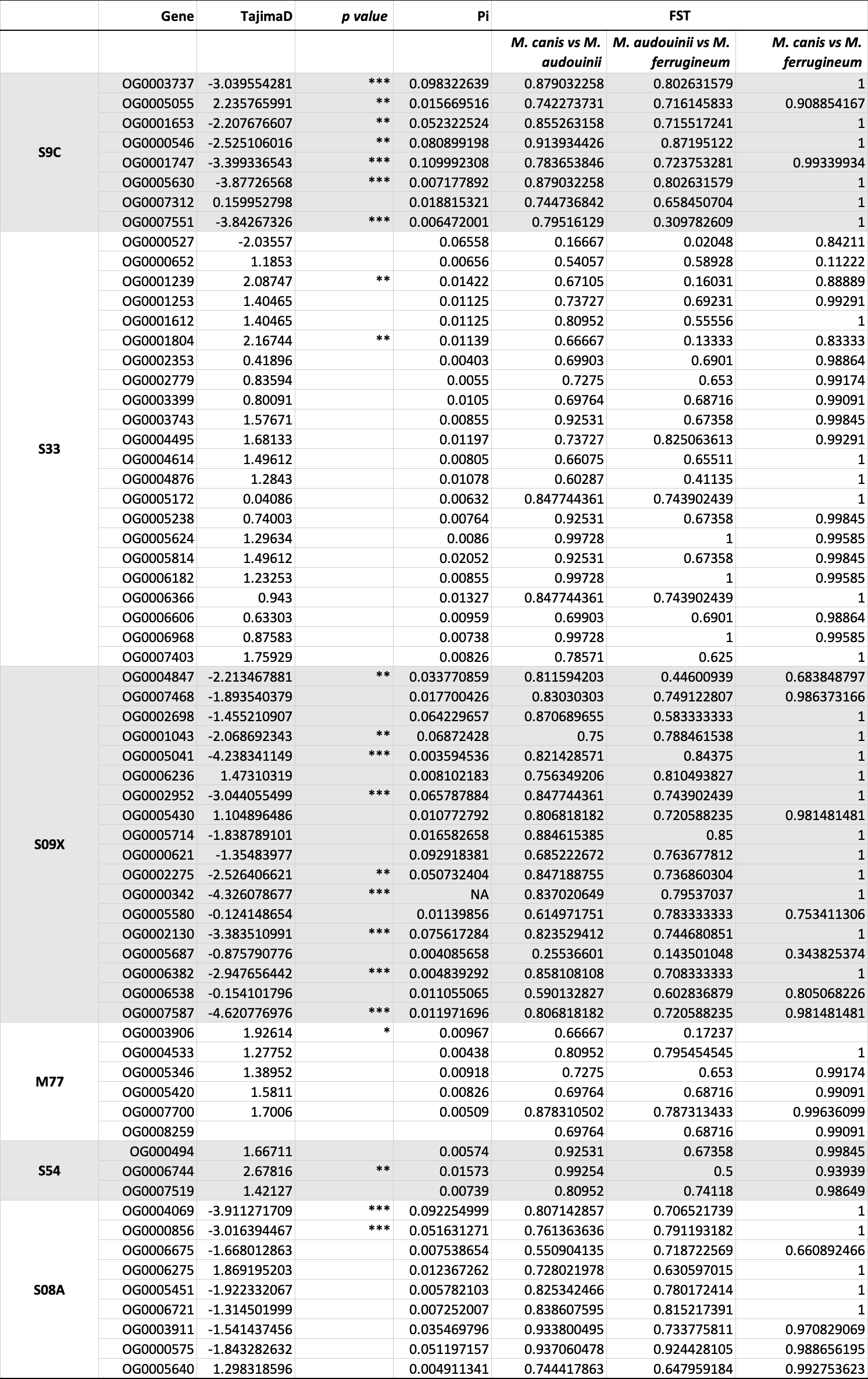
Table S5. Genetic diversity and selection analysis of protease subfamilies in *Microsporum* species.**


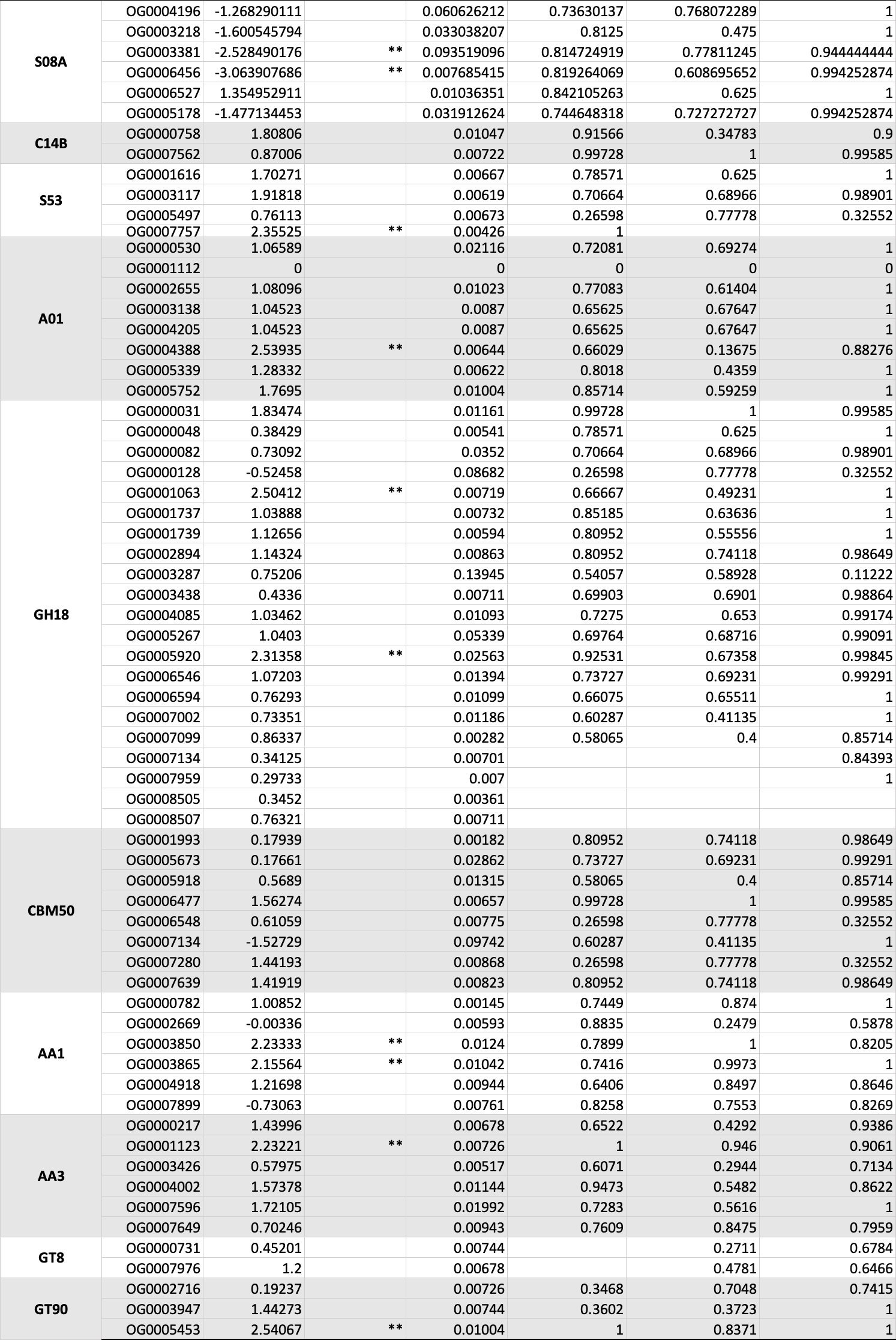
**Table S5. (Continued).**

**References**

Cingolani P, Platts A, Wang le L, Coon M, Nguyen T, Wang L, Land SJ, Lu X, Ruden DM. 2012. A program for annotating and predicting the effects of single nucleotide polymorphisms, SnpEff: SNPs in the genome of Drosophila melanogaster strain w1118; iso-2; iso-3. Fly. 6(2):80-92. doi: 10.4161/fly.19695.

Li H. 2013. Aligning sequence reads, clone sequences and assembly contigs with BWA-MEM. arXiv: Genomics. doi: 10.6084/M9.FIGSHARE.963153.V1.
